# Impacts of obesity on global subclinical left cardiac function represented by CMR-derived myocardial strain, TyG index may be a predictor

**DOI:** 10.1038/s41598-023-43343-z

**Published:** 2023-09-25

**Authors:** Jiajie Mei, Yanhua Li, Jianli Dong, Miaomiao Bai, Yinong Jiang, Xiaofeng Qu, Lili Yin

**Affiliations:** 1 Department of Cardiology, The Second Hospital of Dalian Medical University, No. 467 Zhongshan Road, Shahekou District, Dalian, 116027 China; 2 Department of Radiology, The Second Hospital of Dalian Medical University, No. 467 Zhongshan Road, Shahekou District, Dalian, 116027 China; 3 International Medical Department, The Second Hospital of Dalian Medical University, No. 467 Zhongshan Road, Shahekou District, Dalian, 116027 China; 4 Department of Cardiology, First Affiliated Hospital of Dalian Medical University, Dalian, 116011 Liaoning China

**Keywords:** Cardiology, Endocrinology, Risk factors

## Abstract

Obesity is a recognized risk factor for heart failure. People with similar weights may have different metabolic health. Notably, insulin resistance is a hallmark of obesity and a feature of heart failure. We aimed to evaluate the effects of obesity and metabolic health status on subclinical left cardiac function. We also investigated whether insulin resistance (TyG index) plays a role in BMI-linked subclinical left cardiac dysfunction. The study involved 403 volunteers. Hierarchical multiple regression models were used to assess associations between obesity, metabolic health, and overall subclinical left cardiac function. Mediating analysis was used to explore the role of the TyG index in the association between BMI and left cardiac function. Finally, ROC analysis was performed to explore the predictive value of the TyG index in subclinical left cardiac dysfunction. The correlation analysis showed that metabolic unhealth increased the risk of subclinical left ventricular (LV) dysfunction; obesity was associated with an increased risk of global left cardiac dysfunction regardless of metabolic health status. The TyG index mediated 25% of the associations between BMI and Left atrial (LA) functional parameters. ROC analysis exhibited that the TyG index can be used as a predictor of LA dysfunction (AUC = 0.63), and the optimal cut-off point for the TyG index is 9.33. Even a “non-obese metabolically unhealthy” is a detrimental state of early LV function; obesity remains a major risk factor for global subclinical left cardiac dysfunction. Using the TyG index could allow early identification of individuals at high risk of subclinical left cardiac dysfunction.

Registration number: ChiCTR2200057991; Date of registration: 2022-03-25. URL: http://www.chictr.org.cn/showproj.aspx?proj=162316.

## Introduction

Heart failure (HF) is a life-threatening disease and represents the final common pathway for varieties of cardiovascular diseases (CVD). Obesity is a recognized risk factor for cardiovascular disease and HF, and there is a significant, dose-dependent connection between body mass index (BMI) and the risk of overall HF, as demonstrated by prior research^1^.

However, although BMI may be a convenient and simple indicator for monitoring the increase in the prevalence of obesity at the population level, studies have shown that obesity, as defined by BMI, is significantly heterogeneous^2^, and even at the same BMI level, Asian individuals are predisposed towards visceral obesity (including increased epicardial fat)^3,4^. Indeed, people with similar weight or BMI values may also have substantially different metabolic health^5^. In addition, metabolic unhealth and visceral obesity are also cardiometabolic risk phenotypes, fitting well with the concept of a lipodystrophy-like phenotype in the general population^6^.

It has been suggested that insulin resistance is a plausible mediator in the progression from normal weight to obesity and, eventually, gradual metabolic deterioration^7^. Notably, insulin resistance is a hallmark of obesity and a feature of HF^8^. A Mendelian randomization study confirmed that genetically instrumented higher insulin resistance was associated with a higher risk of HF^9^. Recently, the triglyceride–glucose (TyG) index, calculated from fasting triglyceride and glucose, has been proven to have a powerful linkage with hyperinsulinaemia-euglycemia clamping (the gold standard for insulin resistance), which can be used as a convenient, economical and reliable surrogate indicator of insulin resistance^10^. A recent analysis of the ARIC study revealed that elevated levels of the TyG index are significantly related to left ventricular dysfunction and an increased risk of failure^11^.

Left atrial (LA) strain has been shown to incrementally predict cardiovascular events particularly, but not exclusively, in patients with HF with preserved ejection fraction (HFpEF)^12^. Assessment of LA strain allows earlier detection of LA dysfunction^13^. Similarly, The global longitudinal strain (GLS) of the left ventricle (LV) is a novel biomarker of heart function tested for risk stratification in HF^14^. A recent mesa study^15^ indicates that the left atrioventricular coupling index (LACI), which was defined by the ratio between the LA end-diastolic volume (LAVmin) and the LV end-diastolic volume(LVEDV), and a higher LACI reflecting a greater impairment of left atrioventricular coupling, has improved prognostic value in predicting HF events compared with traditional HF factors.

Currently, the impact of obesity and metabolic unhealthy conditions on subclinical cardiac dysfunction is still unclear. Hence, we aimed to evaluate the effects of different obesity and metabolic health status on subclinical global left cardiac function (including LA function, atrioventricular coupling, and LV function) in participants without prevalent cardiovascular disease. Further, we also analyzed whether the TyG index (an indicator of insulin resistance) plays a role in BMI-linked subclinical left cardiac dysfunction.

## Materials and methods

### Study population

We recruited 403 consecutive asymptomatic participants who were in good financial standing, aged 20–80 years old, and did not have a history of organic diseases such as severe heart disease, stroke, renal insufficiency, malignant tumors, etc. Some participants suffer from metabolic disorders such as high blood pressure, diabetes, obesity, etc. To promote heart health comprehensively, participants voluntarily undergo blood tests, cardiac ultrasounds, cardiac magnetic resonance (CMR), and other related examinations, between April 2022 and December 2022. Additionally, participants who self-reported specific medications (e.g. diabetes/antihypertensive drugs) in the past 1 month were excluded from the study to minimize interference with the results. Females of reproductive age and postmenopausal were included in the study.

All subjects provided informed consent to participate in this study before enrollment. This study was approved by the Ethics Committee of the Second Affiliated Hospital of Dalian Medical University and complied with the principles of the Declaration of Helsinki. A written informed consent form was obtained from each subject.

### Data collection and definitions

All participants were given a medical evaluation consisting of clinical history and physical examination, including anthropometrics and blood pressure (BP) measurement. Fasting venous blood samples were collected on the same day as the echocardiographic and CMR examinations.

Hypertension was defined as systolic blood pressure ≥ 140 mm Hg or diastolic blood pressure ≥ 90 mm Hg. Diabetes mellitus (DM) was defined by a fasting glucose ≥ 126 mg/dl. HOMA-IR was calculated as fasting insulin (µU/ml) × fasting blood glucose (mg/dl)/405, TyG index as ln[fasting triglyceride (mg/dL) × fasting glucose (mg/dL)/2]^11^, and mean arterial pressure (MAP) as diastolic BP + 1/3(systolic BP—diastolic BP). Obesity was defined according to the standard BMI published by the World Organization (WHO) (Asian cutoff 25 kg/m^2^)^16^, and participants were divided into 2 groups based on BMI, obese as 25 kg/m^2^ and non-obese as 18–24.9 kg/m^2^.

Further, we constructed a metabolic score (MS) as the sum of the z-transformed values of the TyG index, MAP, and total cholesterol (TC). This score was then dichotomized at the value of 1, resulting in a group of metabolically healthy (MH) individuals (i.e., MS < 1) and a group of metabolically unhealthy (MU) individuals (i.e., MS ≥ 1), an approach introduced previously^17^.

### Conventional echocardiography

Each patient underwent an echocardiography examination, which was measured with a Vivid E9 ultrasound system (GE Vingmed Ultrasound, Horten, Norway). The mitral inflow velocity was obtained in the apical four-chamber view by pulsed-wave Doppler echocardiography during early (E) and late filling (A), and the E/A ratio was calculated. The sample volume was placed at the lateral mitral annulus to measure the myocardium's early diastolic velocity (e′)^18^.

### MRI analysis

#### MRI protocol

All MR measurements were performed using a 3.0-T whole-body scanner (Skyra; Siemens Medical Solutions, Erlangen, Germany) with a dedicated 32-channel body phased-array coil for when patients are in the supine position. Cardiac dimensions and function were assessed with ECG-triggered balanced steady-state free precession cine sequences. Cine images including three long-axis views (2-chamber, 3-chamber, 4-chamber) and 2-chamber short-axis views were acquired (slice thickness: 8.0 mm; field of view: 360 × 300 mm; matrix size: 256 × 166 pixels; flip angle: 40°; repetition time: 2.81 ms; and echo time: 1.22 ms)^18^.

### Imaging analysis

#### LA and LV volumetric and functional analysis

Cardiac magnetic resonance (CMR) can provide accurate morphological and functional LA and LV estimation in a ‘one-stop-shop’ scan. Two experienced radiologists performed the image postprocessing on commercial software (CVI42; Circle Cardiovascular Imaging, Inc., Calgary, Canada). The images were analyzed blind to the individuals’ basic personal information (including age, sex, etc.). Firstly, the endocardial contour of the LA and LV was manually delineated in the end-diastolic and end-systolic images of the LV. Additionally, the long and short diameters of the LA were measured. An LA volumetric analysis was performed according to the biplane area length method. The system automatically calculates the maximum LA volume (LAVmax) and minimum LA volume (LAVmin). Then, LV parameters were calculated, including LV end-diastolic volume (LVEDV), LV ejection fraction (EF), and LV mass (LVM), which were also automatically computed according to the standardized image interpretation and post-processing. The LVM was indexed for BSA to determine the LVM index (LVMI)^18^.

#### LA and LV strain analysis

Tissue tracking technology was used to track each myocardial voxel on the horizontal 4-chamber long-axis and vertical 2-chamber long-axis cine slices. Then the software automatically analyzed the LA strain indices. The strain and strain rate (SR) values were averaged across three tracking studies. The LA strain parameters included reservoir strain (LAs-s, which corresponds to atrial reservoir function at LV end-systole), conduit strain (LAs-e, which corresponds to atrial conduit function during early diastole), and booster strain (LAs-a, which corresponds to atrial booster function during late diastole).

LV strain parameters could be obtained using the same software and a similar tracking method. The LV endocardial and epicardial borders were manually drawn in the 2-chamber, 4-chamber, and short-axis views of the end-diastolic and end-systole phases. The LV strain indexes, including longitudinal strain (GLS), were obtained by tracking the long horizontal axis cines, whereas the circumferential (GCS) and radial strain (GRS) were derived from the short axis cine^18^.

### Quantification of epicardial adipose tissue (EAT)

EAT is represented by regions of high signal intensity between the myoepicardium and parietal pericardium, semi-automatically traced and calculated after the exclusion of blood vessels. To acquire quantitative EAT, short-axis and long-axis four-chamber cine slices were imported into the tissue char module in CVI 42. EAT volume was measured during the end-diastolic phase on the short-axis cine slices and the single four-chamber view cine images^18^.

### Statistical analysis

All data were analyzed using SPSS statistical software (version 26.0; SPSS Inc., Chicago, IL, USA). Categorical data are presented as numbers (percentages) and were compared using the chi-square. Normally distributed continuous variables are expressed as the mean ± SD and were compared using a one-way analysis of variance (ANOVA). Pearson’s or Spearman’s correlation coefficient applied to the entire study population was used to analyze the bivariable correlations as appropriate.

Hierarchical multiple regression analysis applied to the entire study population was employed to identify the relationship between BMI, EAT, Metabolic score, TyG index and left cardiac functional parameters, and multicollinearity was diagnosed by variance inflation factor (VIF). Further, we performed a mediation analysis to understand the indirect role of TyG index between BMI and subclinical left cardiac dysfunction. The proportion explained by the intermediate factors as follows: 100% × [Beta-coefficient_model_−Beta-coefficient_model+ intermediatefactor_]/[Beta-coefficient_model_]^19^. A two-sided p-value of < 0.05 was considered statistically significant. The current study defined subclinical LA dysfunction as LA reservoir strain < 23%^20^. Receiver operating characteristic curve (ROC) analysis was conducted to predict BMI and TyG index to subclinical LA dysfunction.

### Ethics approval and consent to participate

This study was approved by the institutional ethics of The Second Hospital of Dalian Medical University. All subjects provided written informed consent.

## Results

### Characteristics, cardiac structural and functional parameters of study participants

This study included a total of 403 participants (mean age: 55.71 ± 11.28 years, male sex: 58.6%, mean BMI: 25.49 ± 3.80 kg/m^2^), and the baseline characteristics, cardiac structural and functional parameters are shown in Table 1.

**Table 1:** Characteristics, cardiac structural and functional parameters of study participants.

	Total	Non-obese (n = 209)	Obese (n = 194)	p
Characteristic				
Age, years	55.71 ± 11.28	56.98 ± 11.10	54.43 ± 11.28	0.022
BMI, kg/m^2^	25.49 ± 3.80	22.40 ± 1.84	28.37 ± 2.75	< 0.001
SBP, mmHg	136.52 ± 21.28	134.52 ± 22.13	138.26 ± 20.33	0.078
DBP, mmHg	89.00 ± 12.32	86.97 ± 12.62	90.90 ± 11.75	0.001
Male, n(%)	236 (58.6)	93 (47.9)	143 (68.4)	< 0.001
Any alcohol use, n(%)	32 (7.9)	11 (5.7)	21 (10.0)	0.104
Smoker, n(%)	26.3 (106)	45 (23.2)	61 (29.2)	0.172
Diabetes, n(%)	172 (42.8)	72 (37.3)	100 (47.8)	0.033
Hypertension, n(%)	193 (47.9)	72 (37.1)	121 (57.9)	< 0.001
Laboratory data				
Hs-CRP, mg/L	1.71 ± 3.55	1.19 ± 2.14	2.20 ± 4.45	0.006
BNP, pg/mL	50.41 ± 232.36	61.87 ± 314.75	37.09 ± 39.73	0.417
LDL-C, mmol/L	2.98 ± 0.89	3.04 ± 0.86	2.93 ± 0.92	0.209
HDL-C, mmol/L	1.25 ± 0.31	1.37 ± 0.33	1.13 ± 0.24	< 0.001
TC, mmol/L	5.13 ± 1.08	5.27 ± 1.07	5.01 ± 1.08	0.019
TG, mmol/L	1.81 ± 1.43	1.55 ± 1.05	2.06 ± 1.67	< 0.001
FPG, mmol/L	6.01 ± 2.06	5.70 ± 1.63	6.31 ± 2.35	0.003
Ins, mU/L	10.40 ± 8.16	8.34 ± 9.17	12.11 ± 6.70	< 0.001
eGFR, ml/min/1.73 m^2^	118.90 ± 38.37	114.95 ± 34.19	122.68 ± 41.70	0.044
Others				
HOMA-IR	2.94 ± 2.70	2.23 ± 2.25	3.56 ± 2.91	< 0.001
TyG index	8.85 ± 0.70	8.67 ± 0.67	9.03 ± 0.68	< 0.001
Metabolic score	-0.02 ± 2.01	-0.40 ± 2.03	0.36 ± 1.92	0.001
EAT, ml	47.61 ± 19.22	42.42 ± 17.34	52.80 ± 19.68	< 0.001
Echocardiographic statistics				
E/A	0.94 ± 0.32	0.97 ± 0.36	0.91 ± 0.27	0.034
E/e′	8.55 ± 2.59	8.37 ± 2.51	8.71 ± 2.65	0.315
CMR statistics				
LAVI, ml/m^2^	32.70 ± 12.70	30.96 ± 12.16	34.36 ± 13.03	0.036
LAs-s, %	38.76 ± 13.67	42.03 ± 14.70	35.61 ± 11.84	< 0.001
LAs-a, %	17.16 ± 6.25	18.30 ± 6.56	16.08 ± 5.76	0.005
LAs-e, %	21.44 ± 9.70	23.44 ± 10.51	19.52 ± 8.45	0.001
LV EDV, ml	134.95 ± 28.33	125.97 ± 20.91	143.65 ± 31.75	< 0.001
LVMI, g/m^2^	75.95 ± 21.55	73.71 ± 21.44	78.15 ± 21.5	0.094
EF, %	60.64 ± 7.34	61.88 ± 7.01	59.45 ± 7.48	0.009
LV GRS, %	31.65 ± 7.84	33.28 ± 7.31	30.02 ± 8.05	0.002
LV GCS, %	− 18.40 ± 3.16	− 19.09 ± 2.84	− 17.7 ± 3.32	0.001
LV GLS, %	− 16.42 ± 3.54	− 16.95 ± 2.77	− 15.89 ± 4.12	0.023
LACI, %	20.86 ± 12.14	17.92 ± 9.53	23.83 ± 13.72	< 0.001

Values are mean ± SD, or n (%).

eGFR, estimated glomerular filtration rate; BMI, body mass index; hs-CRP, High-sensitivity C-reactive protein; FPG, fasting plasma glucose; TC, total cholesterol; TG, triglycerides; LDL-C low-density lipoprotein cholesterol; HDL-C high-density lipoprotein cholesterol; BNP, B-type natriuretic peptide; EAT, epicardial adipose tissue; Ins, fasting insulin; SBP, systolic blood pressure; DBP, diastolic blood pressure;LA, left atrial; LV, left ventricular; LAV, LA volume; LAVI, LA volume index; LAs-s, LA reservoir strain; LAs-a, LA booster strain; LAs-e, LA conduit strain; LAsr-s, LA reservoir strain rate; LAsr-a, LA booster strain rate; LAsr-e, LA conduit strain rate; LAEDV, LV end-diastole volume; LVMI, left ventricular mass index; E, the peak early transmitral flow velocity; A, the peak late transmitral flow velocity; e′, the lateral mitral annular velocity; GCS, global circumferential strain; GLS, global longitudinal strain; GRS, global radial strain; LACI, left atrioventricular coupling index; EF, LV ejection fraction.

The estimated glomerular filtration rate (eGFR) and B-type natriuretic peptide (BNP) levels of all participants were within the normal range. In general, obese study participants had a higher prevalence of comorbidities and higher levels of insulin resistance, EAT volume, and metabolic scores (p ≤ 0.001).

In terms of LA and LV structure, it was shown that greater LA(such as LA volume and LA volume index) and LV structural characteristics(such as LV end-diastole volume and LVMI) were observed in obese individuals(p < 0.05). With regards to LA function, all LA functional parameters (LA reservoir, LA conduit, and LA booster strain) were observed to be considerably decreased in obese participants (p < 0.05). Moreover, the LACI was significantly higher in the obese group than in the non-obese group (23.83 ± 13.72 vs 17.92 ± 9.53, p < 0.001). Regarding LV functional parameters, the mean EF of all participants was 60 ± 7.34%, all within the normal range. Additionally, in this study, no differences were observed between obese and non-obese individuals regarding E/e′. As far as left ventricular strain is concerned, all LV strains (including LV GLS, LA GCS, and LV GRS) were reduced in obese individuals (p < 0.05).

### Association between obesity, metabolic health and left cardiac functional parameters

**Figure 1:**
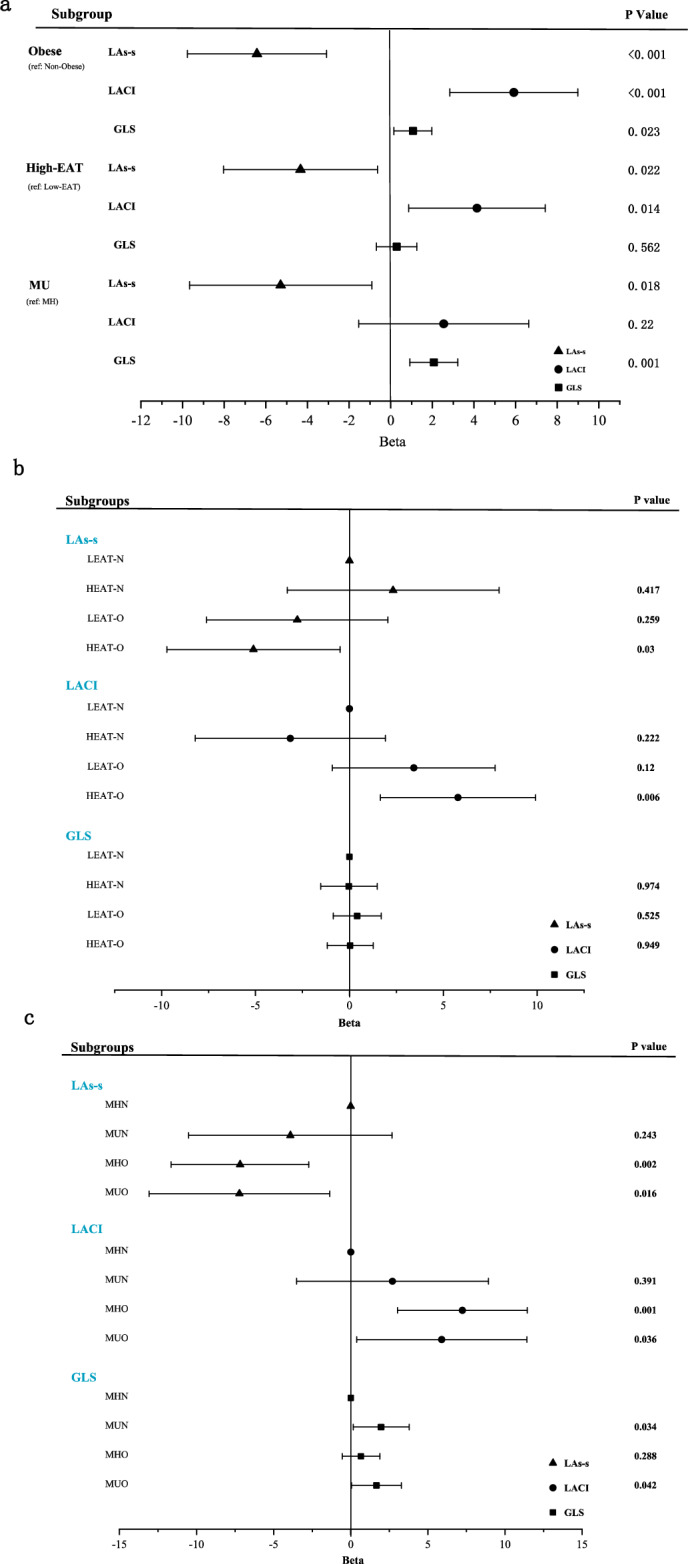
Forest plots depicting multivariable associations between left cardiac function and different obesity and metabolic health status. (**a**) Association between left cardiac function and obesity or metabolic health. (**b**) Combined subgroups of BMI and EAT (LEAT-N group as reference); (**c**) combined subgroups of BMI and metabolic health (MHN group as reference); Model adjusted for age, sex, smoking status, alcohol use, hypertension and diabetes. EAT was analyzed as binary (split by the median EAT volume level)variables. Low-EAT, below the median; high-EAT, above the median; MHN, metabolically healthy and non-obese; MUN, metabolically unhealthy and non-obese; MHO, metabolically healthy and obese; MUO, metabolically unhealthy and obese; LEAT, low EAT volume(below the median);HEAT, high EAT volume(above the median); LEAT-N, combined of low EAT volume and non-obese; HEAT -N, combined of high EAT volume and non-obese; LEAT-O, combined of low EAT volume and obese; HEAT-O, combined of high EAT volume and obese; LAs-s, LA reservoir strain; LACI, left atrioventricular coupling index; GLS, LV global longitudinal strain; Beta coefficients reflect the adjusted HRs for cardiac function of obesity and metabolic health status.

**Figure 2:**
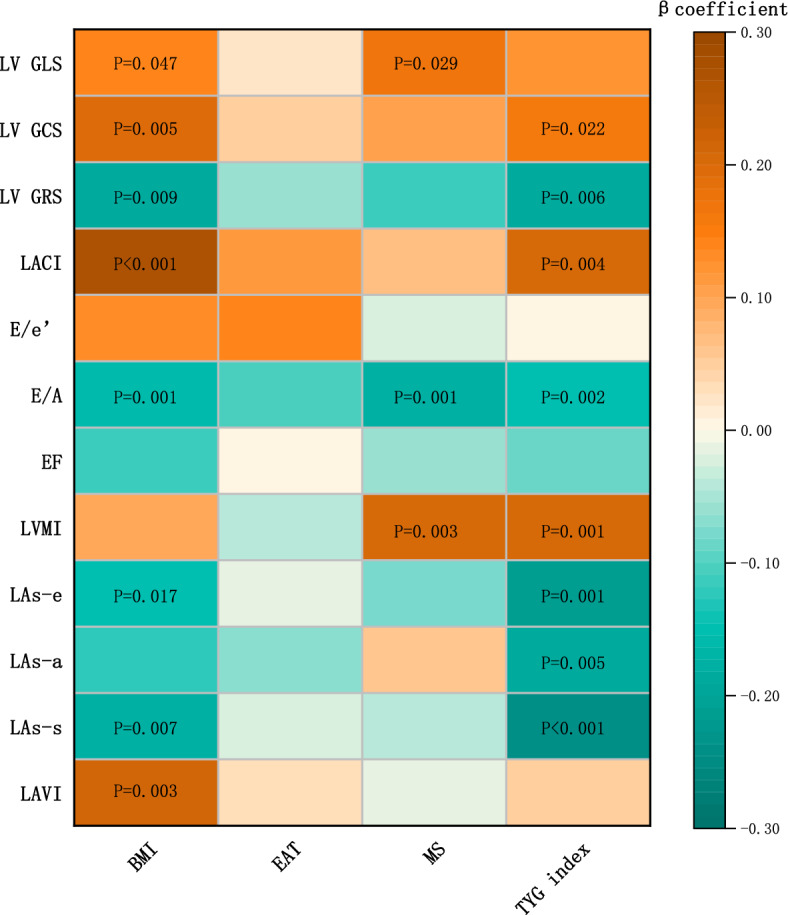
Heatmap illustrating multivariate associations between obesity, metabolic score, TyG index and left cardiac structure and function. The values in the cells represent the P values for the associations. The P value with an insignificant correlation is not shown in the figure. The color and intensity of each cell depicts the standardized β-coefficients from linear regression models adjusted for age, sex, smoking status, alcohol use, hypertension and diabetes. Positive correlations in this figure are highlighted in brown, whereas negative correlations are highlighted in green. LA, left atrial; LV, left ventricular; LAVI, LA volume index; LAs-s, LA reservoir strain; LAs-a, LA booster strain; LAs-e, LA conduit strain; GLS, LV global longitudinal strain; GCS, LV global circumferential strain; GRS, LV global radial strain; LACI, left atrioventricular coupling index; LVMI, left ventricular mass index; EAT, epicardial adipose tissue; MS, metabolic score.

Table 2 shows the correlation between BMI, EAT, Metabolic score, TyG index and left cardiac functional parameters (Models adjusted for age, sex, smoking status, alcohol use, hypertension and diabetes, and the VIF of all independent variables are less than 5). Our analysis focuses on LA reservoir strain (LAs-s), LACI, and LVGLS as the key parameters of left cardiac function, considering their predictive abilities for LA dysfunction and LV dysfunction, respectively^20^. After multivariate adjustment, simple obesity substantially worsens overall left cardiac functional parameters (including LAs-s, LACI, and LV GLS) compared with non-obese individuals (p < 0.05, Fig. 1). Similarly, when BMI was considered a continuous variable, BMI showed a significant association with poorer overall left cardiac functional parameters (p < 0.05, Fig. 2).

**Table 2:** Te correlation between BMI, EAT, Metabolic score, TyG index and left cardiac functional parameters.

	BMI, kg/m^2^		EAT, ml		Metabolic score		TyG index	
	r	p	r	p	r	p	r	p
EAT, ml	0.313	< 0.001	–	–	–	–	–	–
Metabolic score	0.167	0.005	–	–	–	–	–	–
TyG index	0.234	< 0.001	–	–	–	–	–	–
LAVI, ml/m^2^	0.192	0.003	0.028	0.686	− 0.01	0.893	0.097	0.137
LAs-s, %	− 0.175	0.007	− 0.027	0.704	− 0.043	0.562	− 0.238	< 0.001
LAs-a, %	− 0.111	0.087	− 0.069	0.319	0.055	0.46	− 0.182	0.005
LAs-e, %	− 0.155	0.017	− 0.01	0.882	− 0.086	0.251	− 0.218	0.001
LVMI, g/m^2^	0.091	0.143	− 0.046	0.521	0.207	0.003	0.198	0.001
EF, %	− 0.110	0.090	0.006	0.937	− 0.054	0.47	− 0.085	0.192
E/A	− 0.163	0.001	− 0.116	0.103	− 0.192	0.001	− 0.162	0.002
E/e′	0.124	0.058	0.139	0.122	− 0.02	0.793	0.006	0.931
LACI, %	0.250	< 0.001	0.109	0.116	0.062	0.426	0.195	0.004
LV GRS, %	− 0.175	0.009	− 0.057	0.415	− 0.113	0.144	− 0.185	0.006
LV GCS, %	0.187	0.005	0.046	0.512	0.098	0.205	0.155	0.022
LV GLS, %	0.133	0.047	0.022	0.753	0.168	0.029	0.121	0.074

Model adjusted for age, sex, smoking status, alcohol use, hypertension and diabetes.

BMI, body mass index; LAVI, LA volume index; LAs-s, LA reservoir strain; LAs-a, LA booster strain; LAs-e, LA conduit strain;; LVMI, left ventricular mass index; E, the peak early transmitral flow velocity; A, the peak late transmitral flow velocity; e′, the lateral mitral annular velocity; GCS, global circumferential strain; GLS, global longitudinal strain; GRS, global radial strain; LACI, left atrioventricular coupling index; EF, LV ejection fraction.

Based on the previously reported effects of EAT on the myocardium and cardiac function^20,21^, we focus on EAT level as representative of visceral obesity. Compared to low-EAT (EAT volume below the median), high-EAT (EAT volume above the median) increases the risk of impaired LA functional parameters and LACI, but has no substantial impact on LVGLS (Fig. 1). As a continuous variable, EAT was not independently associated with left cardiac functional parameters after multivariate adjustment. (Fig. 2).

Additionally, metabolic unhealth increases the risk of decreased LA functional parameters (LAs-s) and LVGLS, but with little effect on LACI (Fig. 1). Interestingly, metabolic score was only associated with LVGLS, but not LA functional parameters or LACI (Fig. 2).

### Associations between combination of obesity and metabolic health and left cardiac functional parameters

Figure 1 shows the combined effect of obesity and metabolic health on left cardiac functional parameters. Multivariable linear analysis showed that participants with high EAT obesity were at the highest risk of impaired LA functional parameters (adjusted β = − 5.11, p = 0.03) and LACI (adjusted β = 5.78, p = 0.006), whereas no difference in risk was observed for LVGLS, with the low EAT-nonobese group as reference (Fig. 1B).

It is intriguing to note that obesity and metabolic unhealth may have distinct effects on left cardiac performance. In the presence of obesity, LA functional parameters and LACI were more impaired regardless of whether the individuals were metabolically healthy or unhealthy(p < 0.05), and the presence of a combination of obesity and metabolic unhealth significantly increases the risk of impaired GLS, with the MHN(metabolically healthy and non-obese) group as reference. Notably, being metabolically unhealthy (MUN:adjusted β = 1.98, p = 0.03 or MUO:adjusted β = 1.67, p = 0.04), the LV GLS becomes worse regardless of obesity or non-obesity, with the MHN group as a reference (Fig. 1C).

### TyG index as a mediator between BMI and left cardiac function (LA functional parameters)

**Figure 3:**
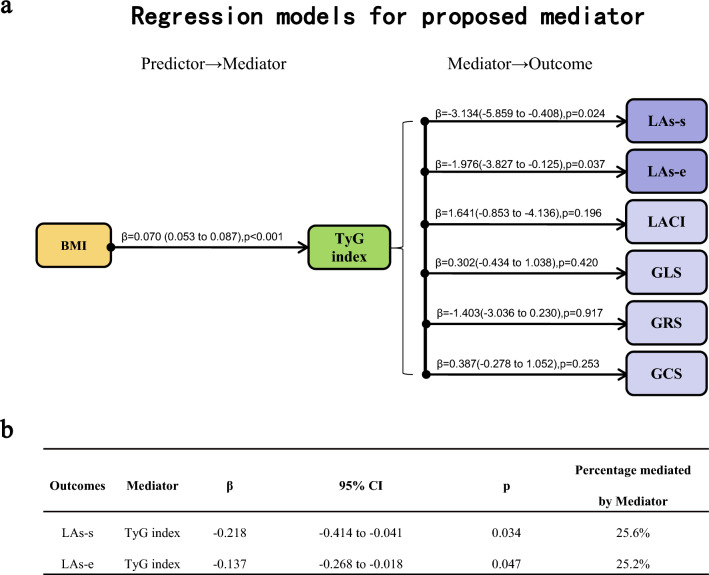
Mediation analysis of TyG index as mediators of the association between BMI and left cardiac function. Values adjacent to the arrows depict β-coefficients (95% CIs) and P values from linear regression models adjusted for age, sex, smoking status, alcohol use, hypertension and T2DM. The total effect of the association of BMI with outcomes (left cardiac function) on linear regression analysis is a prerequisite for mediation analysis. (**a**) Investigates the assumptions that BMI is associated with increased mediators (TyG index) and that mediators are associated with increased/decreased outcomes (LA function, LACI, LV function). Outcomes in light purple do not fulfill the assumptions for mediation analysis because there is no statistically significant effect between mediator and outcome. (**b**) Mediated effect in mediation analysis of parameters indicating that 25.2–25.6% of the association of BMI with decreased LA function is mediated by TyG index. Percent mediated = mediated effect/total effect × 100. Beta coefficients reflect the change in the dependent variable per 1 kg/m^2^ increase in BMI. Abbreviations as in Figs. 1, 2.

All variables that fulfilled a 3-way association between BMI, TyG index, and left cardiac function parameters were entered into formal mediation analyses. It was found that BMI, TyG index, and LA function (but not LV function or LACI) met the 3-way correlation, and thus entered the next mediation analysis. The TyG index mediated 25.2–25.6% of the associations between BMI and LA function indices (LAs-s, LAs-e) (Figs. 2, 3).

### Comparison of ROC curves for the prediction of the LA dysfunction using TyG index and BMI

**Figure 4:**
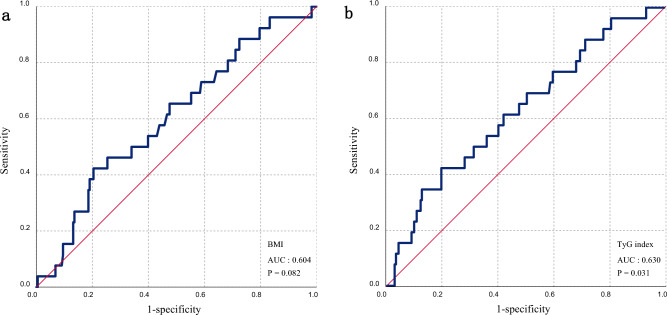
ROC analysis to predict the relationship between the LA dysfunction and BMI (**a**), TyG index (**b**).

In the final analysis, we compared the ROC curves of BMI and the mediator TyG index for predicting LA dysfunction, respectively (Fig. 4). In terms of predicting LA dysfunction, the area under the ROC curve for BMI was 0.60 (p = 0.082) and the area under the ROC curve for the TyG index was 0.630 (p = 0.031). Accordingly, the TyG index can be used as a predictor of LA dysfunction, and the optimal cut-off point for TyG index is 9.33.

## Discussion

This is the first study to evaluate the separate and combined impacts of obesity and metabolic unhealth on subclinical left cardiac function (including LA function, left atrioventricular coupling, and LV function) among participants without overt cardiac disease. In addition, for the first time, this study demonstrates that the TyG index is not only a mediator between BMI and subclinical LA function (rather than LACI or LV function), but may also be a reliable predictor of subclinical LA dysfunction.

Obesity and metabolic unhealth have been major risk factors for cardiovascular disease worldwide^22^. People of normal weight but metabolically unhealthy may have greater than three-fold the risk of cardiovascular events than those of normal weight but metabolically healthy^6^. A recent large-scale cohort study conducted in China revealed that metabolically unhealthy individuals were significantly more likely to develop a variety of vascular diseases than metabolically healthy individuals in the same BMI category, highlighting the significance of individuals of all BMI levels being aware of the risk of metabolic unhealth in the prevention of early cardiovascular events^23^.

In a post hoc analysis of data from the SOS study, blood pressure, dyslipidemia, and hyperglycemia were used as parameters to assess metabolic health and stratify cardiometabolic or mortality risks in obese individuals^24^. Accordingly, the metabolic score (MS), which includes blood pressure, triglycerides, cholesterol, and glucose, was used in this study to assess metabolic health status, which is also the method used in a recent large study^17^. Our study revealed that regardless of obesity status, metabolically unhealthy subjects were observed to have a significantly higher risk of subclinical LV function impairment than metabolically healthy individuals. Furthermore, obesity (as represented by BMI) contributes to overall subclinical left cardiac dysfunction (including LA function, LACI, and LV function) regardless of metabolic health status. The present study adds to previous evidence that even non-obese individuals with metabolic unhealth are not benign, and metabolic unhealth may damage myocardial function, especially LV function, even at the early stage of being asymptomatic. Of great concern, these findings highlight that obesity (rather than metabolic unhealth) is a major risk factor for global subclinical left cardiac function.

BMI is significantly correlated with total body fat content as the optimal tool for measuring obesity^4^, but BMI does not account for the variation in visceral obesity between individuals^25^. In Japan, researchers from the Longitudinal Amagasaki Visceral Fat Study, which classified individuals based not only on BMI but also on the basis of estimated visceral fat levels, showed that variations in BMI and visceral fat were associated with changes in the risk of CVD^26^. According to the International Atherosclerosis Society and the International Chair on Cardiometabolic Risk Working Group on Visceral Obesity, visceral adiposity represents emerging cardiovascular risk factors^4^.

EAT is a unique visceral fat depot that is located between the epicardium and the myocardium, and it has been shown that EAT is more sensitive to lipogenesis than other types of visceral adipose tissue^27^. In addition, EAT has been proposed to contribute to cardiac remodeling and HF^28^. Our study suggested that participants with a combination of high EAT and obesity had the highest risk of impaired subclinical left cardiac function (with low EAT-non-obesity as reference), particularly LA function and LACI. Despite this, EAT was not independently associated with parameters of subclinical left cardiac function, which was in contrast with previous studies^29^. Possible explanations are as follows: first, the subjects in the study were at different stages of the disease or had different risk factors in addition to the disease; second, previous studies have mostly studied EAT thickness through ultrasound, whereas our study used CMR (as gold standard) to study EAT volume; and third, participants are grouped differently in different studies, which may cause differences in the findings. Furthermore, the close relationship between BMI and visceral fat has been confirmed, and EAT volume may depend on BMI level^30^. Overall, we highlighted that obesity represented by BMI was the primary risk factor for subclinical left cardiac function impairment in this study, and high EAT levels contribute to risk stratification for subclinical left cardiac dysfunction.

As it has been proven, further weight gain, the worsening of insulin resistance, and there is an interaction between obesity and insulin resistance^31^. Obesity and insulin resistance increase inflammation and oxidative stress, resulting in endothelial dysfunction and cardiomyocyte apoptosis, thereby reducing myocardial deformation capacity and ultimately damaging myocardium, resulting in varying degrees of decreased myocardial function^32^, and these are important explanations for the decrease in subclinical left cardiac function (myocardial strain) due to obesity.

It is noteworthy that insulin resistance is a hallmark of obesity and a feature of HF^8^. Moreover, recent meta-analysis results suggest a possible causal association between insulin resistance and incident HF^33^. In other studies, insulin resistance also appears to be significantly associated with cardiac remodeling and dysfunction^11^. The hyperinsulinemic-euglycaemic clamp test(as the gold standard) is time-consuming, costly, and invasive, thereby limiting its application in clinical practice^11^. The TyG index(rather than HOMA-IR) has been shown to have a good correlation with the hyperinsulinemic-euglycaemic clamp test^10^. Hence, the TyG index was used in this study to represent insulin resistance and demonstrated that a higher TyG index was associated with a higher BMI and worse global subclinical left cardiac function (including LA function, LACI, and LV function) independent of traditional risk factors. These findings underscored the potential utility of using the TyG index as a potential predictor of cardiac remodeling, making clinicians facilitate the identification of high-risk patients with HF at an early stage.

Our results indicate that insulin resistance can lead to subclinical left atrial dysfunction even in individuals with normal EF. The latest correlation study^34^ also reported early functional alterations of myocardial contractile fibers in normal glucose tolerance patients with one-hour plasma glucose values ≥ 155 mg/dL, even before the reduction in LVEF. In a future prospective study, It could be interesting to evaluate the impact of the TyG index on early cardiac function in different glycometabolic phenotypes.

Several mechanisms have been proposed to contribute to the increased vulnerability of myocardium in insulin resistant states, including increased mitochondrial dysfunction, metabolic alterations in substrate utilization, oxidative stress, bioactive lipid accumulation, inflammation, increased apoptosis, altered calcium metabolism and signaling, increased apoptosis, and myocardial fibrosis^8,31^. In addition, insulin resistance also has a direct adverse effect on the myocardium^33^. Despite this, the mechanisms underlying the association between insulin resistance (as represented by the TyG index) and HF risk are not entirely understood. Further studies are required to figure out the implicated mechanism.

Notably, we also observed that the TyG index mediated the correlation between BMI and subclinical LA function (rather than LV function), as well as serving as an appropriate predictor of LA dysfunction in our study. Recently, the European Association of Cardiovascular Imaging (EACVI) published a consensus document that advocates the use of LA strain for HF with preserved ejection fraction, and LA strain is an integral part of assessing myocardial mechanics and hemodynamics^35^. The LA reservoir function (reservoir strain) has been proved to provide a more accurate categorization of diastolic dysfunction than other cardiac structural and functional variables, such as LV GLS, or E/e′^36^. It has been suggested that lower LA reservoir function may indicate more advanced LA remodeling and underlying LV dysfunction^37^; therefore, the strength of the LA strain lies in the integration of information on both LV and LA function and volume^38^. Furthermore, among the functional indicators examined, LA reservoir strain and conduit strain were the most reliable predictors of HF death or admission^39^. The findings of our study are of great clinical importance for preventing the development of HF among the general population, highlighting the utility of evaluating LA function for risk stratification and recommending the TyG index as a practical indicator for predicting subclinical left cardiac dysfunction in clinical practice.

### Clinical implications

The novelty of this study is to support that a “metabolically unhealthy” state is a relatively deleterious state for subclinical LV function, highlighting the importance of metabolic health in the early prevention of cardiac function injury. Additionally, obesity (as represented by BMI) was a major risk factor for global subclinical left cardiac function impairment, as compared with metabolic unhealth and high EAT levels. A large cohort study has demonstrated that obesity remains a significant risk factor for major vascular diseases in Chinese adults, regardless of metabolic disorders^23^. This study adds to the evidence supporting strategies to prevent obesity in the general Chinese population, regardless of an individual’s metabolic health status. In addition, we recognize that high EAT levels may also contribute to risk stratification for early cardiac dysfunction, emphasizing the importance of focusing on visceral obesity in addition to obesity to further refine the definition of “generalized obesity” to better combat the growing epidemic of obesity worldwide.

It is worth mentioning that our results provide valuable information to elucidate the underlying mechanisms linking insulin resistance and subclinical LA dysfunction. In light of the overwhelming burden of HF worldwide, insulin resistance may be a crucial therapeutic target for early prevention of HF. Furthermore, identifying insulin resistance using the TyG index may allow early identification of high-risk individuals who may benefit from specific interventions for preventing incident HF.

### Study limitation

Several limitations should be noted. Firstly, A potential limitation is the lack of consistency in the definition of metabolic health^40^ when comparing our results with those of previous studies. But even so, previous studies have also stated similar conclusions to this study, which is that obesity remains an independent risk factor for cardiovascular disease, independent of metabolic factors^23,40^. Second, due to the cross-sectional nature of our study, we were unable to establish causality. In spite of the fact that our study controlled for a variety of potential risk factors, including comorbidities, age, sex, smoking, and alcohol consumption, residual confounders may remain of concern, and other factors such as diet or physical activity, may contribute to mediator-outcome confounding as well. Additionally, there is still no consensus among scientists and clinicians regarding the parameters and cut-off values that define metabolic health, and a different, widely accepted definition may be developed in the future. Furthermore, although these data highlight insulin resistance as a predictor of early left atrial dysfunction, there are limitations, and there is no universally accepted cutoff value for insulin resistance. As our study was restricted to Chinese adults without overt cardiac disease, our findings may not be broadly applicable to other ethnic groups and regions, but may still offer a basis for HF prevention in the future.

## Conclusion

Even a “non-obese metabolically unhealthy” state is a detrimental state of early LV function; obesity remains a major risk factor for global subclinical left cardiac dysfunction. EAT is a measurable cardiovascular risk factor that adds some value to the stratification for early cardiac function impairment. Insulin resistance, represented by the TyG index, mediates the correlation between BMI and subclinical left cardiac function, and the use of the TyG index could potentially allow early identification of individuals at high risk for subclinical left cardiac dysfunction.

## Data Availability

The datasets used and/or analyzed during the current study are available from the corresponding author on reasonable request.

## Abbreviations

BMI: Body mass index
TYG: Triglyceride–glucose
ROC: The receiver operating characteristic curve
CMR: Cardiac magnetic resonance imaging
EAT: Epicardial adipose tissue
LAVI: LA volume index
LAs-s: LA reservoir strain
LAs-a: LA booster strain
LAs-e: LA conduit strain
LAsr-s: LA reservoir strain rate
LAsr-a: LA booster strain rate
LAsr-e: LA conduit strain rate
LVMI: Left ventricular mass index
GCS: Global circumferential strain
GLS: Global longitudinal strain
GRS: Global radial strain
LACI: Left atrioventricular coupling index
HOMA-IR: Homeostatic model assessment of insulin resistance
